# Effects of m^6^A methylation of MAT2A mRNA regulated by METTL16 on learning and memory, hippocampal synaptic plasticity and Aβ_1–42_ in 5 × FAD mice

**DOI:** 10.3389/fnagi.2025.1572976

**Published:** 2025-04-16

**Authors:** Huan Chen, Fangzhen Guo, Yan Zhao, Wei Liu, Bingyu Chen, Chang Wang, Lining Huang, Sufang Jiang, Xiaowei Ma, Huiling Ren, Sha Li, Huixian Cui

**Affiliations:** ^1^Department of Human Anatomy, Neuroscience Research Center, Hebei Medical University, Shijiazhuang, China; ^2^Hebei Key Laboratory of Neurodegenerative Disease Mechanism, Shijiazhuang, China; ^3^School of Nursing, Hebei Medical University, Shijiazhuang, China; ^4^Department of Immunology, Hebei Medical University, Shijiazhuang, China; ^5^Department of Anesthesiology, Second Hospital of Hebei Medical University, Shijiazhuang, China; ^6^The Key Laboratory of Clinical Neurology, Ministry of Education, Shijiazhuang, China; ^7^Department of Neurology, First Hospital of Hebei Medical University, Shijiazhuang, China; ^8^Department of Neurology, Third Hospital of Hebei Medical University, Shijiazhuang, China; ^9^The Key Laboratory of Neural and Vascular Biology, Ministry of Education, Shijiazhuang, China

**Keywords:** Alzheimer’s disease, m^6^A methylation modification, METTL16, MAT2A, learning and memory

## Abstract

**Background:**

Alzheimer’s disease (AD) is a common neurodegenerative disorder affecting older adults, characterized by progressive cognitive decline and pathological features such as amyloid plaque deposition, neuronal loss, and synaptic reduction. RNA N6-methyladenosine (m^6^A) methylation is prevalent in the brain and is intricately linked to synaptic plasticity, learning, and memory in AD. However, the precise mechanisms underlying these associations remain elusive.

**Methods:**

This study employed the overexpression of methyltransferase-like protein 16 (METTL16), or overexpression of methionine adenosyltransferase 2A (MAT2A), or a combination of METTL16 overexpression with MAT2A knockdown to explore the influence of METTL16 on the regulation of MAT2A in cognitive function, hippocampal synaptic plasticity, and amyloid-beta (Aβ_1–42_) metabolism in 5 × FAD mice.

**Results:**

Our findings indicated a reduction in m^6^A methylation levels and the expression of METTL16 and MAT2A in the hippocampus of 5 × FAD mice. Overexpression of METTL16 led to an increase in overall m^6^A methylation levels, furthermore, overexpression of either METTL16 or MAT2A enhanced learning and memory in 5 × FAD mice, elevated the expression levels of postsynaptic density 95 (PSD95) and synaptophysin (Syp), increased dendritic spine density, and decreased the accumulation of Aβ_1–42_ in the hippocampus. In the hippocampus of 5 × FAD mice, METTL16 was found to upregulate both the protein and mRNA levels of MAT2A, as well as enhance MAT2A mRNA m^6^A methylation levels. Concurrent, overexpression of METTL16 and knockdown of MAT2A in the hippocampus resulted in impaired learning and memory in 5 × FAD mice, alongside a reduction in synaptic protein expression and dendritic spine density, and an increase in Aβ_1–42_ accumulation.

**Conclusion:**

The present study demonstrated that METTL16 enhances learning and memory in 5 × FAD mice by regulating MAT2A mRNA m^6^A methylation, which leads to increased expression levels of PSD95 and Syp, greater dendritic spine density, and reduced Aβ_1–42_ accumulation in the hippocampus. These findings reveal a novel approach for investigating the pathophysiological role of METTL16 in AD and offer new insights for developing of potential therapeutic targets for AD.

## 1 Introduction

Alzheimer’s disease (AD) is one of the most prevalent neurodegenerative disorders worldwide, and is characterized by a gradual onset in older adults and manifested clinically as progressive cognitive decline. The pathological hallmarks of AD primarily include amyloid β-protein (Aβ) deposition, neuronal loss, neurofibrillary tangles, and a reduced number of synapses (Lane et al., 2018; Grobler et al., 2023; Khezri and Ghasemnejad-Berenji, 2023). Synaptic dysfunction in AD, characterized by decreased dendritic spine density and reduced expression of synaptic proteins such as postsynaptic density 95 (PSD95) and synaptophysin (Syp), manifests years before the appearance of overt clinical symptoms and is strongly correlated with the severity of memory impairments (Bertoni-Freddari et al., 1990; de Pins et al., 2019; Han et al., 2024; Jack and Holtzman, 2013; Long et al., 2021; Masliah et al., 2001). These findings highlight synaptic plasticity as a crucial therapeutic target for AD intervention.

N6-methyladenosine (m^6^A) represents the most prevalent form of RNA methylation modification within the eukaryotic transcriptome, is abundantly present across various tissues (Liufu et al., 2024) and plays a significant role in RNA nuclear export, stability, splicing, translation, and subcellular localization (Zhang et al., 2019). It is intimately linked with numerous neurological disorders (Zhang N. et al., 2022; Han et al., 2020). Recent evidence underscores the importance of epigenetic modifications, particularly m^6^A, in the regulation of synaptic plasticity and cognitive functions. For example, m^6^A methylation mediated by methyltransferase-like protein 3 (METTL3) and METTL14 is critical for synaptic plasticity, while the m^6^A demethylase fat mass and obesity-associated protein (FTO) modulates memory consolidation by modulating m^6^A levels (Zhang et al., 2018; Koranda et al., 2018; Walters et al., 2017; Li et al., 2018). The dysregulation of the m^6^A machinery, exemplified by deficiencies in YTH N6-methyladenosine RNA-binding protein F1 (YTHDF1) or the upregulation of circRNA regulating synaptic be exocytosis 2 (circRIMS2), exacerbates hippocampal synaptic defects and cognitive impairments in AD models (Shi et al., 2018; Wang X. et al., 2023). Recent studies intriguingly suggest that m^6^A plays a role in AD pathogenesis through mechanisms involving tau hyperphosphorylation and Aβ metabolism, thereby establishing it as a critical epigenetic regulator in neurodegeneration (Li et al., 2018; Yin et al., 2023).

METTL16 operates in both the nucleus and cytoplasm, participating in RNA biogenesis, decay, and translation processes (Su et al., 2022; Ruszkowska, 2021; Nance et al., 2020; Satterwhite and Mansfield, 2022). Our previous research demonstrated that METTL16 stabilizes methionine adenosyltransferase 2A (MAT2A) mRNA via m^6^A methylation, thereby enhancing MAT2A protein expression and supporting synaptic plasticity in murine models (Zhang R. et al., 2022). MAT2A, a pivotal enzyme in the methionine cycle, regulates the synthesis of S-adenosylmethionine (SAM), a universal methyl donor essential for methylation reactions, including m^6^A modification. This interaction suggests a potential feedback loop among METTL16, MAT2A, and m^6^A dynamics in maintaining neuronal homeostasis.

Despite significant advancements in the field, the role of METTL16 in the pathogenesis of AD remains insufficiently explored. Considering the well-documented associations between m^6^A dysregulation and AD-related synaptic dysfunction, we propose that a deficiency in METTL16 may disrupt MAT2A-dependent SAM production, thereby impairing m^6^A homeostasis and exacerbating Aβ pathology, synaptic loss, and cognitive deficits characteristic of AD. This study aims to elucidate the mechanisms by which METTL16 modulates MAT2A activity to influence AD pathology, learning, and memory, thereby offering a novel perspective on the pathophysiological role of METTL16 in AD and identifying potential therapeutic targets.

## 2 Methods

### 2.1 Animals

Male C57BL/6J mice (Fukang Biological Technology Co., Ltd. China) and 5 × FAD mice (JAX Labs, United States), aged 5 and 6 months were kept at a 12-h light/dark cycle at a constant temperature of 21–22°C, with unrestricted access to food and water. All animal care and experimental procedures were conducted in accordance with the Guidelines for the Management and Use of Experimental Animals and received approval from the Animal Experiment Ethics Committee of Hebei Medical University (IACUC#2021070).

### 2.2 RNA m^6^A quantification

Following isoflurane anesthesia, the hippocampus was dissected, and total RNA was extracted using an RNA extraction kit (cat#: ZP404, ZOMANBIO, China). Subsequent to RNA quantification, the EpiQuik m^6^A RNA methylation quantitative assay kit (cat#: P-9005, Epigentek, United States) was used to assess the m^6^A levels in accordance with the manufacturer’s protocol. Negative and positive controls at varying concentrations, along with 200 ng of sample RNA, were each added to the binding solution and incubated at 37°C for 90 min. Post-incubation, the samples were washed, and a capture antibody, detection antibody, and enhancer solution were added, mixed, and incubated for 1 h. Following another wash step, a developer was added and mixed. After incubation in the dark for 10 min, a stop solution was introduced to terminate the enzymatic reaction. Approximately 40 s later, the liquid in the positive control well turned yellow, and the absorbance was measured at 450 nm using a microplate reader (Molecular Devices, Sunnyvale CA, United States).

### 2.3 Western blotting

Following isoflurane anesthesia, the hippocampus was dissected, and RIPA lysate was added to the sample, which was then disrupted by sonication. The protein was subsequently extracted and quantified. Following denaturation, the proteins underwent sodium dodecyl sulfate-polyacrylamide gel electrophoresis and were subsequently transferred onto polyvinylidene fluoride membranes. The membranes were blocked for 1 h before being incubated overnight at 4°C with primary antibodies: anti-METTL16 (cat#: ab252420, Abcam, United States), anti-MAT2A (cat#: NBP1-92100, Novus, United States), anti-PSD95 (cat#: ab18258, Abcam, United States), anti-Syp (cat#: CY5273, Abways, China), GAPDH (cat#: AB0036, Abways, China). Subsequently, the membranes were incubated with rat anti-mouse (cat#: 18-4417-32, Rockland, United States) and goat anti-rabbit fluorescent secondary antibodies (cat#: 611-145-002, Rockland, United States) for 2 h, protected from light. Finally, the membranes were then visualized using the Odyssey Infrared Laser Scanning Imaging System (LICOR, United States) and analyzed with ImageJ software. For quantitative analysis, each protein band was equal-area delineated using the rectangular selection tool to measure the integrated density of the target proteins and GAPDH. The integrated density of GAPDH served as a reference to determine the relative expression levels of the target proteins.

### 2.4 Stereotaxic injection of virus

AAV vectors carrying the mCherry reporter gene were engineered to overexpress either METTL16 or MAT2A, with an empty vector AAV serving as the control. A volume of 0.5 μL of AAV suspension was administered into the hippocampus of 5-month-old mice via stereotaxic brain injection under isoflurane anesthesia. Additionally, an AAV vector carrying the EGFP reporter gene was constructed to facilitate MAT2A knockdown, with an empty vector AAV as the control. Equal volumes of AAV-oe-METTL16 were combined with either AAV-sh-MAT2A or the vector control. Subsequently, a 1 μL aliquot of the mixed AAV suspension was injected into the hippocampus of 5-month-old mice using stereotaxic brain injection under isoflurane anesthesia. The injection coordinates were set at ML = ±2.46 mm, AP = −2.18 mm, DV = 1.67 mm. Following the establishment of stable AAV expression, neurobehavioral experiments were conducted. The expression of EGFP and mCherry was subsequently examined using fluorescence microscopy to verify the precision of the microinjection site.

### 2.5 Novel object recognition test

Two identical objects were positioned at the left and right extremities of one side wall of an open field box. Mice were then introduced into the box with the two objects oriented towards the rear, allowing them to explore freely for 5 min. After an interval of either 2 or 24 h, one of the objects was substituted with a novel object, and the time spent freely exploring time for both objects was recorded within 5 min. The discrimination index, defined as the ratio of time spent exploring the novel object to the total exploration time, was subsequently calculated.

### 2.6 Y-maze test

In the Y-maze (YM), the three arms were randomly designated as the novel arm, starting arm, and other arm. The novel arm was initially obstructed with a partition, and the mice were placed in the starting arm, permitted to explore freely for 5 min. After 4 h, the partition was removed, and the mice were reintroduced into the starting arm for another 5-min exploration period. A SMART video-tracking system was used to record the time and distance required for exploration of the novel arm.

### 2.7 Morris water maze test

The Morris water maze (MWM) was divided into four quadrants, with a platform of 10 cm in diameter placed in one of the quadrants, designated as the target quadrant. Water was introduced into the pool to submerge the platform by 1 cm, and titanium dioxide powder was added to make the water opaque. Mice were placed into the water from four quadrants for five consecutive days, allowing them to explore freely for 1 min. The escape latency was defined as the time taken by the mice to locate the platform within 1 min. On the sixth day, the platform was removed, and the mice were introduced into the water at the quadrant opposite to where the platform was previously located. The time spent by the mice in the target quadrant and the number of times they crossed the former platform location within 1 min were recorded.

### 2.8 qRT-PCR

Total RNA was extracted from the mouse hippocampus using an RNA extraction kit (cat#: ZP404, ZOMANBIO, China) following the manufacturer’s instructions. The concentration and purity of the RNA were determined by optical density measurement, after which reverse transcription was performed using a cDNA synthesis kit (cat#: R323, Vazyme, China). cDNA amplification was conducted using the ChamQ Universal SYBR qPCR Master Mix system (cat#: Q711, Vazyme, China). The expression of target genes was analyzed using the −ΔΔCT method. The mRNA expressions of METTL16, MAT2A, PSD95, and Syp were analyzed with β-actin serving as the control. The primers used are listed in Table 1.

**Table 1:** The primers used in qRT-PCR.

Name	Primer
METTL16	Forward: 5′-GACAAACCACCTGACTTCGCA-3′
	Reverse: 5′-TCTGACTGCTTCGGGGTCTT-3′
MAT2A	Forward: 5′-GCTTCCACGAGGCGTTCAT-3′
	Reverse: 5′-AGCATCACTGATTTGGTCACAA-3′
PSD95	Forward: 5′-TACCAAAGACCGTGCCAACG-3′
	Reverse: 5′-CGGCATTGGCTGAGACATCA-3′
Syp	Forward: 5′-GCCACTGACCCAGAGAACAT-3′
	Reverse: 5′-TCCTTGAACACGAACCACAG-3′
β-actin	Forward: 5′-TCATCACTATTGGCAACGAGCGGT-3′
	Reverse: 5′-GTGTTGGCATAGAGGTCTTTACG-3′

### 2.9 Golgi staining

Following isoflurane anesthesia, the mice underwent transcardial perfusion with 4% paraformaldehyde. The brains were subsequently dissected and post-fixed in the same fixative for 24 h. They were then immersed in a mixed fixative solution and stored in the dark for 14 days. Following this, the brains were dehydrated in a 30% sucrose solution at 4°C for 2 days. Brain slices with a thickness of 100 μm were prepared using a concussion microtome and incubated with a staining solution for 30 min. Subsequently, a chromogenic solution was added, and the samples were incubated in the dark for an additional 30 min. After undergoing gradient alcohol dehydration and xylene transparency, grade 2 or 3 apical dendrites of neurons in the CA1 region of the hippocampus were observed and photographed using a microscope at 100× magnification. Fiji software was used to analyze images of 30-μm long dendrite segments to calculate dendritic spine density.

### 2.10 ELISA

The mouse Aβ_1–42_ ELISA kit (cat#: CSB-E10787m, CUSABIO, China) was equilibrated for 30 min, after which the standard product or test sample was added to the bottom of the enzyme plate well and incubated at 37°C for 2 h. Subsequently, a biotin-labeled antibody solution and a horseradish peroxidase-labeled avidin solution were introduced to each well for incubation at 37°C for 1 h, followed by the addition of the substrate solution, which was conducted in the absence of light at 37°C for 30 min. Within 5 min, the optical density of each well was measured at 450 nm using a microplate reader (Molecular Devices, United States).

### 2.11 Anti-m^6^A-RIP

Total RNA was extracted utilizing an RNA extraction kit (cat#: ZP404, ZOMANBIO, China). Subsequently, RNA samples, RNA fragment buffer, and nuclease-free water were added sequentially, and the reaction was terminated after incubation at 94°C for 3 min. The RNeasy Micro kit (cat#: 74004, QIAGEN, Germany) was used to purify the RNA. The RNA was then added to the RIP lysis buffer, followed by the addition of RNase inhibitor, m^6^A antibody (cat#: 238003, Synaptic Systems, Germany), and Dynabeads Protein A/G (cat#: 10015D, Invitrogen, United States), and the mixture was incubated at 4°C for 2 h. After washing, the Dynabeads were added to the RIP lysis buffer dissolved with N6-methyladenosine and incubated at 55°C for 30 min. MAT2A mRNA levels were quantified using qRT-PCR.

### 2.12 Statistical analysis

All data were analyzed using SPSS 25 statistical software (IBM Corp., Armonk, NY, United States), and results were expressed as the mean ± SD. The Kolmogorov–Smirnov normality test and Levene’s test for homogeneity of variance were conducted on all experimental results. A *t*-test or one-way analysis of variance was performed on the experimental data, which exhibited a normal distribution (*p* > 0.1) and homogeneity of variance (*p* > 0.1). A significance level of *p <* 0.05 was employed to determine statistical significance.

## 3 Results

### 3.1 Effect of METTL16 on m^6^A methylation modification in hippocampus of 5 × FAD mice

**Figure 1:**
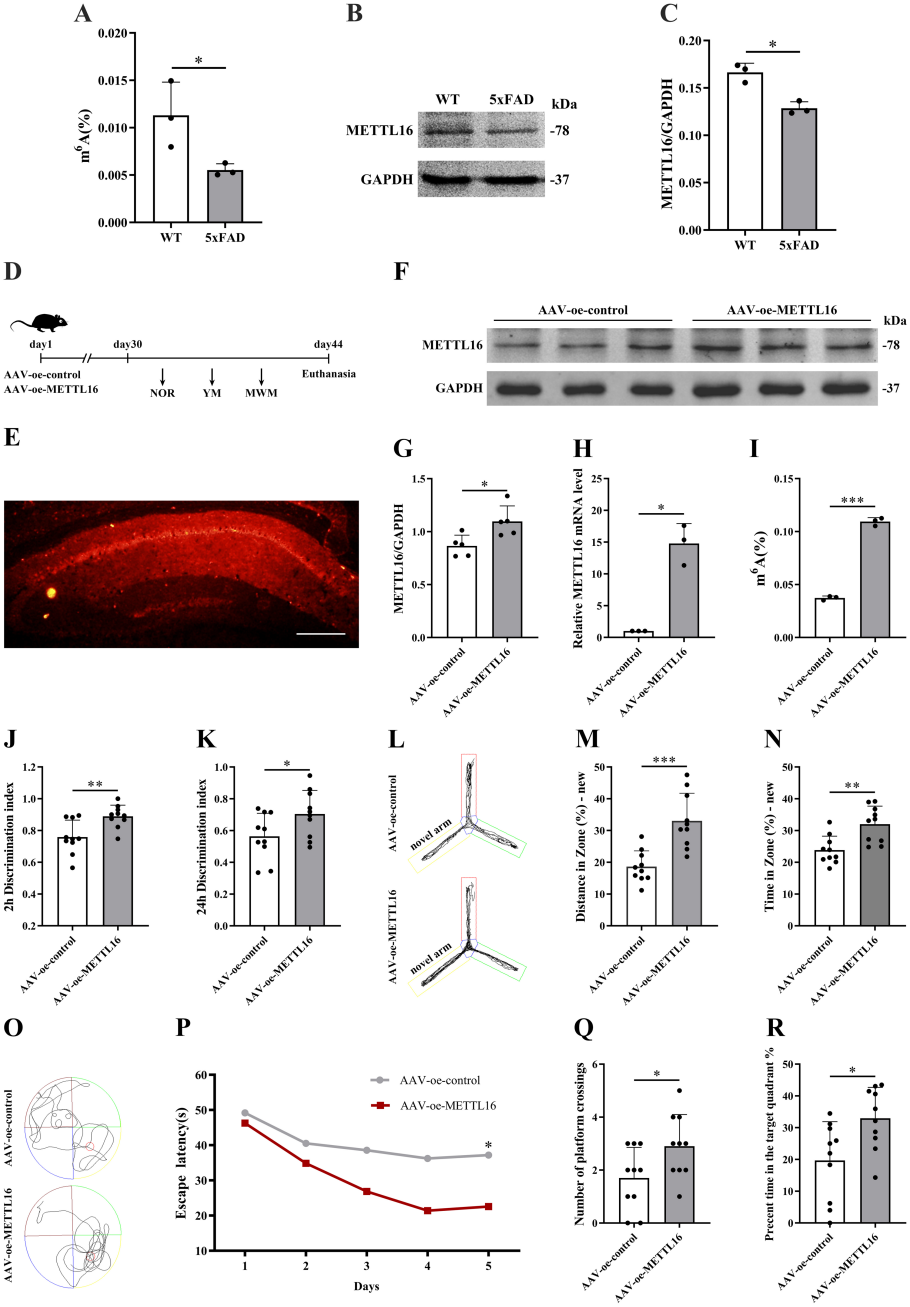
Effects of METTL16 on hippocampal m^6^A methylation modification and learning and memory in 5 × FAD mice. **(A)** m^6^A methylation in the hippocampus of 5 × FAD mice (*n* = 3). **(B,C)** Expression of METTL16 in the hippocampus of 5 × FAD mice (*n* = 3). **(D)** Flow chart illustrating the injection of the overexpressed METTL16 virus and subsequent neurobehavioral experiments. **(E)** Schematic representation of stereoscopic fluorescence diffusion in the hippocampal brains of 5 × FAD mice (scale bar = 500 μm). **(F,G)** Expression level of METTL16 protein after overexpression of METTL16 in 5 × FAD mice (*n* = 5). **(H)** Expression level of METTL16 mRNA after overexpression of METTL16 in 5 × FAD mice (*n* = 3). **(I)** Expression of m^6^A methylation after overexpression of METTL16 in 5 × FAD mice (*n* = 3). **(J,K)** NOR was performed to assess recognition memory after overexpression of METTL16 in 5 × FAD mice (*n* = 10). **(L–N)** YM was performed to assess spatial memory after overexpression of METTL16 in 5 × FAD mice (*n* = 10). **(O–R)** The MWM test was performed to assess spatial memory after overexpression of METTL16 in 5 × FAD mice (*n* = 10). Data are shown as the mean ± SD. ^*^p < 0.05, ^**^p < 0.01, and ^***^p < 0.001.

The expression levels of m^6^A methylation and METTL16 protein in the hippocampus of 5 × FAD mice were assessed using western blotting and RNA m^6^A quantification techniques. The findings demonstrated that both the overall m^6^A methylation level and METTL16 expression in the hippocampus of 5 × FAD mice were reduced compared to WT mice (Figures 1A–C). Subsequently, METTL16 was overexpressed, and the overall m^6^A methylation level in the hippocampus of 5 × FAD mice was evaluated. Figure 1D illustrates the procedure for viral injection and subsequent neurobehavioral experiments. Fluorescence imaging confirmed the accurate localization of AAV-oe-METTL16 in the hippocampus, as shown by its spread (Figure 1E). Results from western blotting and qRT-PCR analyses indicated an increase in METTL16 protein and mRNA expression (Figures 1F–H). Quantification of RNA m^6^A revealed that METTL16 overexpression led to an elevated overall methylation level of m^6^A in the hippocampus of 5 × FAD mice (Figure 1I).

### 3.2 Effects of METTL16 on learning and memory, hippocampal synaptic plasticity and Aβ_1–42_ expression in 5 × FAD mice

**Figure 2:**
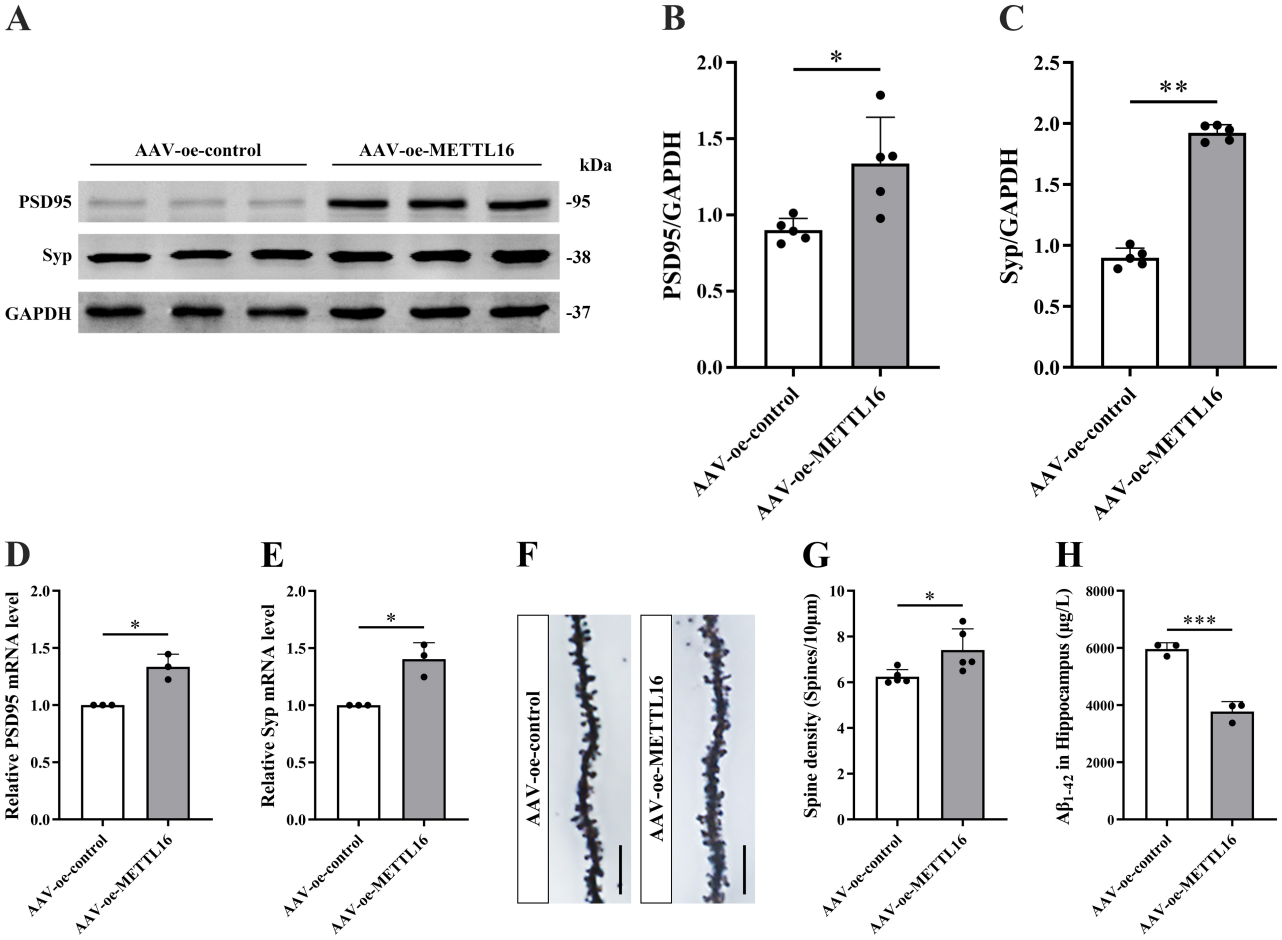
Effects of METTL16 on hippocampal synaptic plasticity and Aβ_1–42_ in 5 × FAD mice. **(A–C)** Expression level of PSD95 and Syp proteins after overexpression of METTL16 in 5 × FAD mice (*n* = 5). **(D,E)** Expression level of PSD95 and Syp mRNA after overexpression of METTL16 in 5 × FAD mice (*n* = 3). **(F)** Golgi staining of hippocampal CA1 region neurons after METTL16 overexpression in 5 × FAD mice (scale bars = 5 μm). **(G)** Quantification of dendritic spine density in hippocampal CA1 region neurons of mice calculated as the number of spines per 10 μm of dendrite (*n* = 5). **(H)** Expression level of Aβ_1–42_ after overexpression of METTL16 in 5 × FAD mice (*n* = 3). Data are shown as the mean ± SD. ^*^p < 0.05, ^**^p < 0.01, and ^***^p < 0.001.

To investigate the potential association between the AD phenotype and methylation modification, changes in learning and memory, hippocampal synaptic plasticity, and Aβ_1–42_ expression levels were assessed in 5 × FAD mice following METTL16 overexpression. The results from the novel object recognition (NOR) test indicated that METTL16 overexpression enhanced the discrimination index at both 2 and 24 h in 5 × FAD mice (Figures 1J,K), suggesting an increased preference for novel object. Furthermore, the YM test results revealed that METTL16 overexpression significantly increased the exploration distance percentage and exploration time percentage in the novel arm of 5 × FAD mice (Figures 1L–N). Additionally, the MWM test results showed that METTL16 overexpression reduced the escape latency of 5 × FAD mice, while increasing both the number of platform crossings and the percentage of exploration time in the target quadrant (Figures 1O–R). The findings from western blotting and qRT-PCR analyses indicated a significant upregulation in the protein and mRNA expression levels of PSD95 and Syp in the hippocampus of 5 × FAD mice following METTL16 overexpression (Figures 2A–E). Golgi staining analysis demonstrated a significant increase in the density of dendritic spines of the CA1 region of the hippocampus in 5 × FAD mice following METTL16 overexpression (Figures 2F,G). ELISA results indicated that METTL16 overexpression led to a reduction in the expression level of Aβ_1–42_ in the hippocampus of 5 × FAD mice (Figure 2H). In summary, METTL16 overexpression enhanced m6A methylation levels, prevented learning and memory deficits, improved hippocampal synaptic plasticity, and reduced Aβ_1–42_ expression in the hippocampus of 5 × FAD mice.

### 3.3 Effects of MAT2A on learning and memory, hippocampal synaptic plasticity, and Aβ_1–42_ expression in 5 × FAD mice

**Figure 3:**
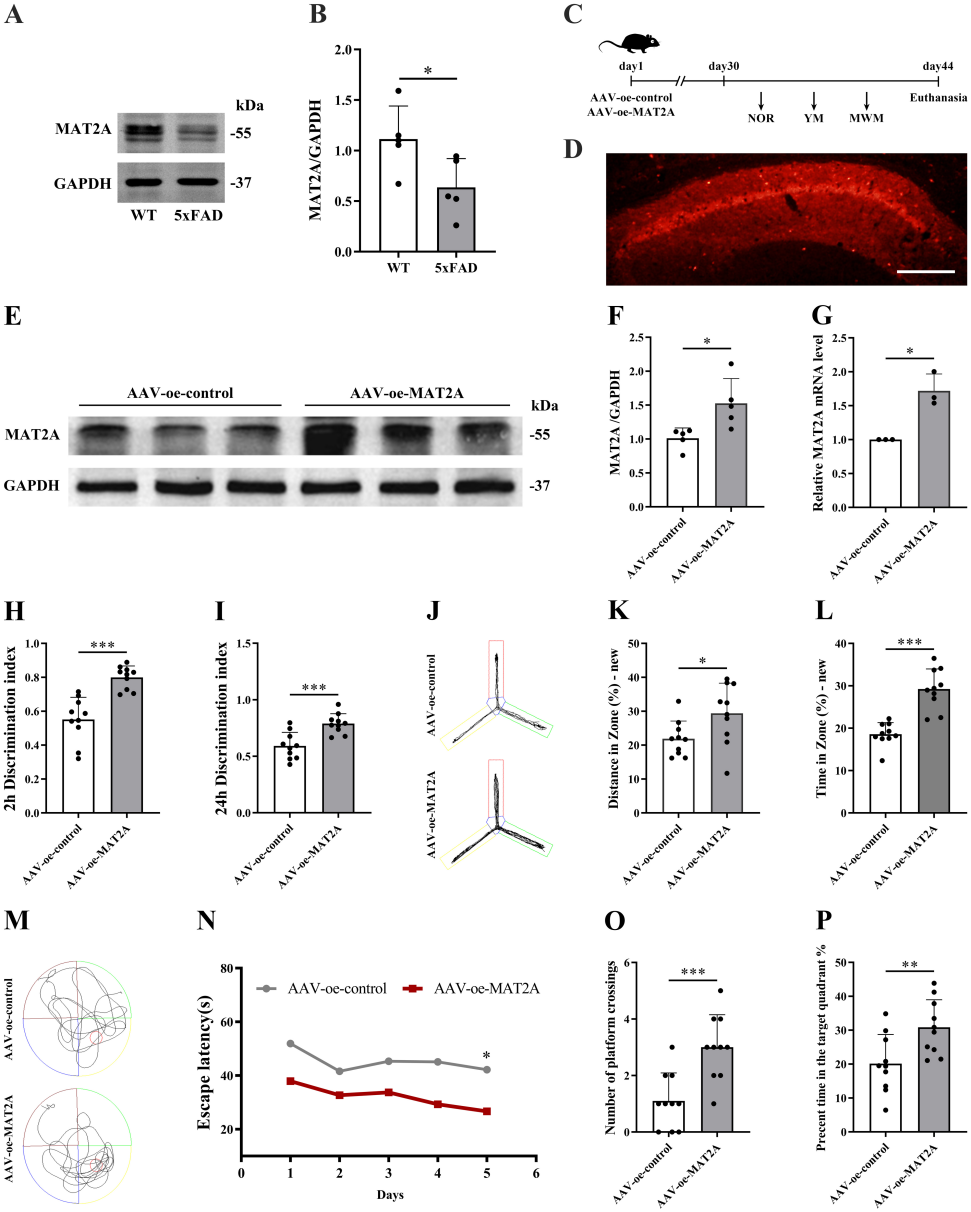
Effects of MAT2A on learning and memory in 5 × FAD mice. **(A,B)** Expression of MAT2A protein in the hippocampus of 5 × FAD mice (*n* = 5). **(C)** Flow chart of injection of overexpressed MAT2A virus and subsequent neurobehavioral experiments. **(D)** Schematic representation of stereoscopic fluorescence diffusion in 5 × FAD mice hippocampal brain (scale bar = 500 μm). **(E,F)** Expression level of MAT2A protein after overexpression of MAT2A in 5 × FAD mice (*n* = 5). **(G)** Expression level of MAT2A mRNA after overexpression of MAT2A in 5 × FAD mice (*n* = 3). **(H,I)** NOR was performed to assess recognition memory after overexpression of MAT2A in 5 × FAD mice (*n* = 10). **(J–L)** YM was performed to assess spatial memory after overexpression of MAT2A in 5 × FAD mice (*n* = 10). **(M–P)** MWM was performed to assess spatial memory after overexpression of MAT2A in 5 × FAD mice (*n* = 10). Data are shown as the mean ± SD. ^*^p < 0.05, ^**^p < 0.01, and ^***^p < 0.001.

The MAT2A protein modulates the overall level of m^6^A methylation by regulating the methyl donor S-adenosylmethionine (SAM). Consequently, we assessed MAT2A expression in the hippocampus of 5 × FAD mice. The results revealed a decrease in MAT2A protein levels in the hippocampus of 5 × FAD mice compared to WT mice (Figures 3A,B).

**Figure 4:**
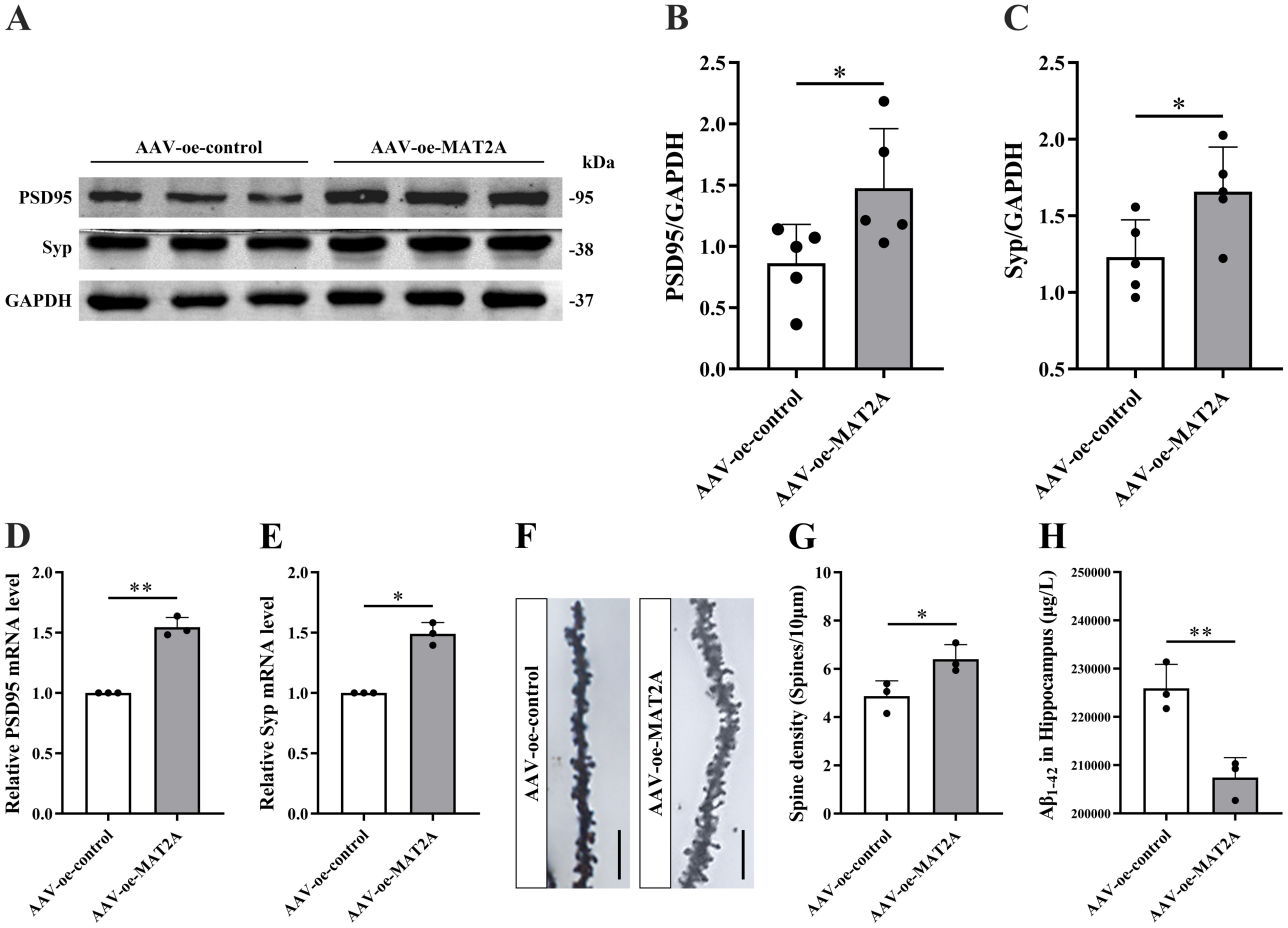
Effects of MAT2A on hippocampal synaptic plasticity and Aβ_1–42_ in 5 × FAD mice. **(A–C)** Expression level of PSD95 and Syp proteins after overexpression of MAT2A in 5 × FAD mice (*n* = 5). **(D,E)** Expression level of PSD95 and Syp mRNA after overexpression of MAT2A in 5 × FAD mice (*n* = 3). **(F)** Golgi staining of hippocampal CA1 region neurons after MAT2A overexpression in 5 × FAD mice (scale bars = 5 μm). **(G)** Quantification of dendritic spine density in hippocampal CA1 region neurons of mice calculated as the number of spines per 10 μm of dendrite (*n* = 3). **(H)** Expression level of Aβ_1–42_ after overexpression of MAT2A in 5 × FAD mice (*n* = 3). Data are shown as the mean ± SD. ^*^p < 0.05 and ^**^p < 0.01.

Subsequently, following MAT2A overexpression, we examined alterations in learning and memory, hippocampal synaptic plasticity, and Aβ_1–42_ expression levels in 5 × FAD mice. The methodology for viral injection and subsequent neurobehavioral experiments is illustrated in Figure 3C. Fluorescence imaging demonstrated that the AAV-oe-MAT2A vector successfully disseminated to the hippocampus, confirming the precision of the injection site (Figure 3D). Western blotting and qRT-PCR analyses revealed elevated levels of MAT2A protein and mRNA expression (Figures 3E–G). The NOR test results indicated that MAT2A overexpression led to an increased discrimination index at both 2 h and 24 h in 5 × FAD mice (Figures 3H,I), indicating an enhanced preference for novel object. In the YM test, MAT2A overexpression resulted in a significant increase in the percentage of exploration distance and exploration time in the novel arm for 5 × FAD mice (Figures 3J–L). MWM test demonstrated that MAT2A overexpression reduced the escape latency in 5 × FAD mice, while increasing both the frequency of platform crossings and the percentage of exploration time in the target quadrant (Figures 3M–P). Furthermore, western blotting and qRT-PCR analyses showed a significant upregulation of PSD95 and Syp protein and mRNA expression levels in the hippocampus of 5 × FAD mice following MAT2A overexpression (Figures 4A–E). Golgi staining revealed a significant increase in the density of neuronal dendritic spines within the CA1 region of the hippocampus in 5 × FAD mice following MAT2A overexpression (Figures 4F,G). Additionally, ELISA results indicated that MAT2A overexpression led to a reduction in the expression level of Aβ_1–42_ in the hippocampus of 5 × FAD mice (Figure 4H). In summary, MAT2A overexpression resulted in enhanced m^6^A methylation levels, prevented learning and memory deficits, improved hippocampal synaptic plasticity, and reduced Aβ_1–42_ expression in the hippocampus of 5 × FAD mice.

### 3.4 Effects of METTL16 regulating MAT2A on learning and memory, hippocampal synaptic plasticity and Aβ_1–42_ expression in 5 × FAD mice

**Figure 5:**
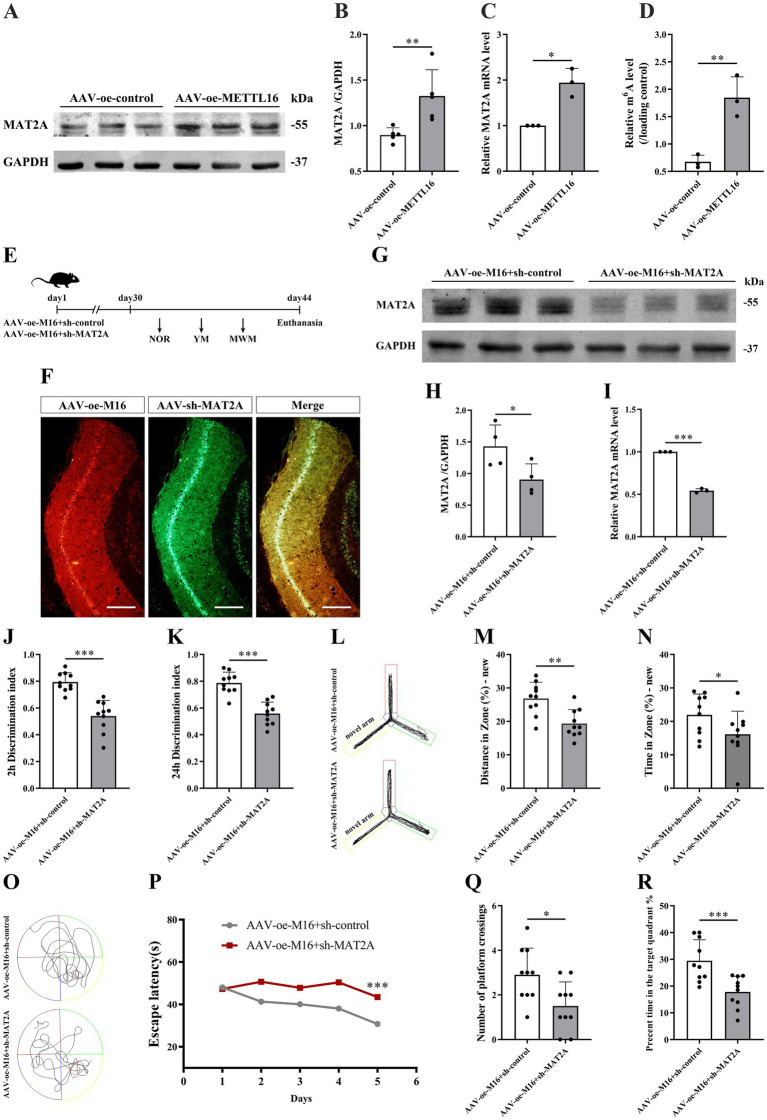
Effects of METTL16 regulation of MAT2A on learning and memory in 5 × FAD mice. **(A,B)** Expression of MAT2A protein in the hippocampus of 5 × FAD mice after METTL16 overexpression (*n* = 5). **(C)** Expression of MAT2A mRNA in the hippocampus of 5 × FAD mice after METTL16 overexpression (*n* = 3). **(D)** m^6^A methylation level of MAT2A mRNA in the hippocampus of 5 × FAD mice after METTL16 overexpression (*n* = 3). **(E)** Flow chart illustrating injection of overexpressed METTL16, knockdown MAT2A virus, and subsequent neurobehavioral experiments. **(F)** Schematic representation of stereoscopic fluorescence diffusion in 5 × FAD mice hippocampal brain (scale bar = 500 μm). **(G,H)** Expression level of MAT2A protein after overexpression of METTL16 and knockdown of MAT2A in 5 × FAD mice (*n* = 4). **(I)** Expression level of MAT2A mRNA after overexpression of METTL16 and knockdown of MAT2A in 5 × FAD mice (*n* = 3). **(J,K)** NOR was performed to assess recognition memory after overexpression of METTL16 and knockdown of MAT2A in 5 × FAD mice (*n* = 10). **(L–N)** YM was performed to assess spatial memory after overexpression of METTL16 and knockdown of MAT2A in 5 × FAD mice (*n* = 10). **(O–R)** MWM was performed to assess spatial memory after overexpression of METTL16 and knockdown of MAT2A in 5 × FAD mice (*n* = 10). Data are shown as the mean ± SD. ^*^p < 0.05, ^**^p < 0.01, and ^***^p < 0.001.

Previous studies have identified MAT2A as a substrate of METTL16. To explore the impact of METTL16 on the expression levels of MAT2A protein and mRNA as well as mRNA m^6^A methylation levels, we conducted western blotting, qRT-PCR, and anti-m6A RIP assays. The findings demonstrated that METTL16 overexpression not only elevated the expression levels of MAT2A protein and mRNA in the hippocampus of 5 × FAD mice (Figures 5A–C) but also increased the m^6^A methylation level of MAT2A mRNA (Figure 5D).

**Figure 6:**
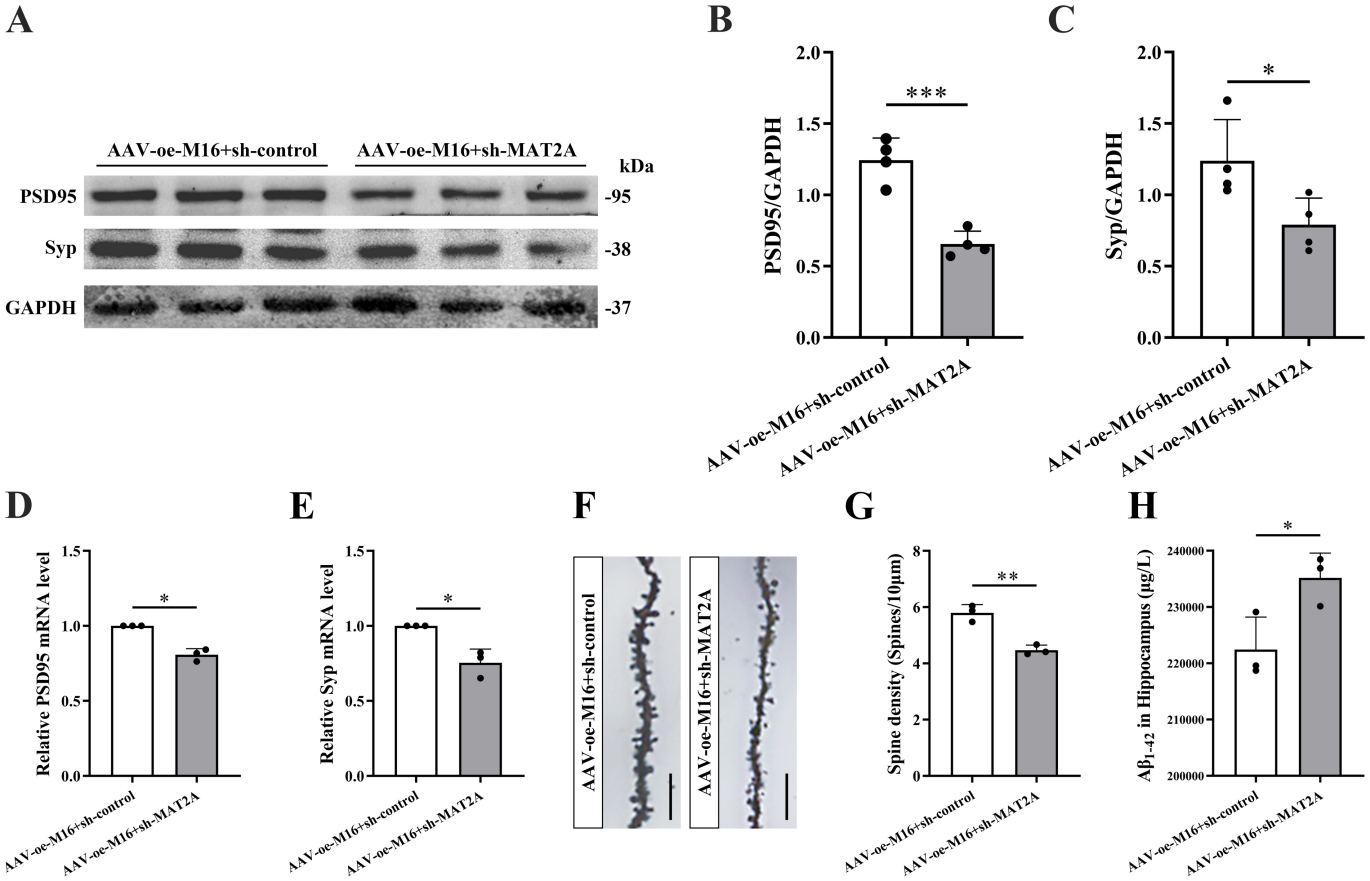
Effects of METTL16 regulating MAT2A on hippocampal synaptic plasticity and Aβ_1–42_ in 5 × FAD mice. **(A–C)** Expression level of PSD95 and Syp proteins after overexpression of METTL16 and knockdown of MAT2A in 5 × FAD mice (*n* = 4). **(D,E)** Expression level of PSD95 and Syp mRNA after overexpression of METTL16 and knockdown of MAT2A in 5 × FAD mice (*n* = 3). **(F)** Golgi staining of hippocampal CA1 region neurons after overexpression of METTL16 and knockdown of MAT2A in 5 × FAD mice (scale bars = 5 μm). **(G)** Quantification of dendritic spine density in hippocampal CA1 region neurons of mice calculated as the number of spines per 10 μm of dendrite (*n* = 3). **(H)** Expression level of Aβ_1–42_ after overexpression of METTL16 and knockdown of MAT2A in 5 × FAD mice (*n* = 3). Data are shown as the mean ± SD. ^*^p < 0.05, ^**^p < 0.01, and ^***^p < 0.001.

Subsequently, we investigated the alterations in learning and memory, hippocampal synaptic plasticity, and Aβ_1–42_ expression in 5 × FAD mice following the concurrent overexpression of METTL16 and knockdown of MAT2A. The methodologies for viral injection and subsequent neurobehavioral assessments are depicted in Figure 5E. Fluorescence imaging confirmed that AAV-oe-METTL16 and AAV-sh-MAT2A effectively disseminated to the hippocampus, verifying the precision of the injection site (Figure 5F). Western blotting and qRT-PCR analyses revealed an increase in MAT2A protein and mRNA expression (Figures 5G–I). The results of the NOR test indicated that the overexpression of METTL16 coupled with the knockdown of MAT2A resulted in a decreased discrimination index at both 2 h and 24 h in 5 × FAD mice (Figures 5J,K), suggesting a diminished preference for novel object. Furthermore, the results from the YM test demonstrated a significant reduction in both exploration distance percentage and exploration time percentage in the novel arm of 5 × FAD mice following METTL16 overexpression and MAT2A knockdown (Figures 5L–N). MWM test results indicated that the overexpression of METTL16 combined with the knockdown of MAT2A in 5 × FAD mice led to an increase in escape latency, alongside a reduction in both the number of platform crossings and the percentage of exploration time within the target quadrant (Figures 5O–R). Western blotting and qRT-PCR analysis demonstrated a significant decrease in the protein and mRNA expression levels of PSD95 and Syp in the hippocampus of 5 × FAD mice subjected to METTL16 overexpression and MAT2A knockdown (Figures 6A–E). Golgi staining further revealed a marked reduction in the density of dendritic spines in the CA1 region of the hippocampus in these mice (Figures 6F,G). ELISA results showed that the expression level of Aβ_1–42_ in the hippocampus was elevated following the same genetic modifications (Figure 6H). Collectively, these findings suggest that the overexpression of METTL16, when coupled with MAT2A knockdown, prevented the beneficial effects of METTL16 overexpression alone on learning and memory, hippocampal synaptic plasticity and Aβ_1–42_ expression in 5 × FAD mice.

## 4 Discussion

Epigenetic regulation plays a crucial role in various neurological disorders, with RNA methylation representing a predominant form of epigenetic modification, constituting approximately 60% of all RNA modifications (Wang X. et al., 2023). RNA dysmethylation implicated in numerous biological processes. Empirical studies have demonstrated that RNA m^6^A methylation is integral to neurogenesis (Wang et al., 2018), learning and memory (Wang X. et al., 2023), brain development (Ma et al., 2018), and axon regeneration (Weng et al., 2018). Moreover, RNA m^6^A methylation is critical for synaptic function (Merkurjev et al., 2018), with alterations in synaptic function being key contributors to the occurrence of AD (Terry et al., 1991). Research has identified that RNA m^6^A methylation influences the pathogenesis of AD in murine models by modulating the expression of hippocampal synaptic proteins (Zhao et al., 2021). Consequently, elucidating the role of m^6^A methylation in AD is essential for advancing our understanding of its pathogenesis.

The m^6^A modification process involves three categories of functional proteins: methyltransferases, demethylases, and recognition proteins. Methyltransferases include METTL3, METTL14, and METTL16 (Jiang et al., 2021); demethylases encompass FTO and alkylation repair homolog protein 5 (ALKBH5) (Gao et al., 2024); and recognition proteins comprise YTH domain-containing proteins 1 and 2 (YTHDC1/2) and YTHDF1/2/3 (Shi et al., 2018; Zálešák et al., 2024). m^6^A methylation and its associated enzymes are critical in modulating neuronal function, as well as in the regulation of learning and memory processes. Specifically, METTL3-mediated m^6^A RNA methylation has been shown to directly influence the formation of long-term memory. A reduction in METTL3 expression levels is associated with an increased risk of AD (Yang et al., 2023). Differential alterations in METTL3 expression have been documented in the hippocampal and cortical regions of APP/PS1 mice (Huang et al., 2022). Furthermore, Koranda et al. (2018) demonstrated that the deletion of the METTL14 protein in mouse striatal neurons led to enhanced neuronal excitability and impaired learning and memory. In our previous study, we observed a significant increase in METTL16 protein expression of in the hippocampus of mice following learning and memory training. The loss of METTL16 resulted in decreased in m^6^A methylation levels, which in turn led to learning and memory impairments and diminished synaptic plasticity (Zhang R. et al., 2022). This was the first study to suggest a role for METTL16 in memory formation and synaptic plasticity within the hippocampus. The process of m^6^A demethylation mediated by ALKBH5 is observed at active synaptic ribosomes and synapses characterized by short-term plasticity. Additionally, there is an increased co-localization of YTHDF1, YTHDF3, and ALKBH5 with m^6^A-modified RNA at activated glutamatergic postsynaptic sites (Martinez De La Cruz et al., 2021). These findings underscore the critical role of m^6^A methylation in sustaining learning and memory functions in mice. In the present study, we observed a reduction in m^6^A methylation and METTL16 expression levels in the hippocampus of 5 × FAD mice. Following the overexpression of METTL16, there was an enhancement in m^6^A methylation levels, accompanied by improvements in learning and memory, increased expression of PSD95 and Syp, elevated dendritic spine density in the CA1 region, and a reduction in Aβ_1–42_ levels in the hippocampus of 5 × FAD mice. These results indicate that modifications in METTL16 expression can lead to significant changes in m^6^A methylation within the hippocampus, thereby exerting a substantial impact on the maintenance of learning, memory and synaptic plasticity stability in mice, corroborating our previous findings. While virus manipulation and vector interference were effectively controlled in this study, the absence of a control group consisting of mice without AAV virus injection limits the ability to thoroughly assess the adverse effects of the virus on the hippocampus and to ascertain whether the biological effects of the virus were restricted by the test genotype. Currently, both the control and experimental groups were subjected to standardized procedures, including consistent virus dosage and postoperative detection, thereby minimizing operational interference. Despite these controls, the observed significant differences between the groups suggest that the phenotype was influenced by the intervention of the target gene, independent of any non-specific effects from the empty vector.

In contrast to METTL3 and METTL14, which require complex formation to catalyze target mRNA methylation, METTL16 primarily operates as a monomer (Satterwhite and Mansfield, 2022). MAT2A is identified as one of the target mRNAs of METTL16 (Pendleton et al., 2017). Notably, this study discovered a reduction in the expression level of the MAT2A protein in the hippocampus of 5 × FAD mice. In contrast, previous studies have demonstrated conflicting results regarding the impact of METTL16 on MAT2A protein expression. Nance et al. (2020) observed that the immediate knockout of METTL16 in cells did not alter MAT2A protein expression. Conversely, Shima et al. (2017) reported a reduction in MAT2A protein levels following the competitive inhibitor of MAT, alongside METTL16 knockdown. These discrepancies may be attributed to variations in the experimental models employed or the duration of viral activity.

Furthermore, Li et al. (2019) discovered that elevated m^6^A methylation levels in adult hippocampal neurons enhance synaptic plasticity, while Zhang et al. (2018) suggested that diminished m^6^A methylation levels in brain tissue may contribute to learning and memory impairments in AD. The potential influence of MAT2A on learning and memory, hippocampal synaptic plasticity and Aβ_1–42_ expression in 5 × FAD mice has not been previously documented. In the present study, we observed that MAT2A overexpression ameliorated learning and memory deficits in 5 × FAD mice, increased the expression of synaptic proteins PSD95 and Syp, and enhanced dendritic spine density, while concurrently reducing Aβ_1–42_ expression levels in the hippocampus. The findings indicate that MAT2A plays a crucial role in learning, memory, and synaptic plasticity, as well as in the expression of Aβ_1–42_ in mice.

In the context of METTL16 functioning as a methyltransferase, SAM serves as a methyl donor, with MAT2A being the principal enzyme responsible for SAM production (Cantoni and Durell, 1957). Previous research has shown that METTL16 influences SAM levels in human embryonic kidney cells by modulating MAT2A RNA (Pendleton et al., 2017). Our previous study demonstrated that METTL16 in the hippocampus enhances the expression of MAT2A protein by stabilizing MAT2A mRNA, thereby increasing the methylation level of m^6^A, which in turn promotes synaptic plasticity and improves learning and memory (Zhang R. et al., 2022). Building on these findings, the present study assessed the impact of METTL16 overexpression on the expression of MAT2A mRNA and protein, as well as the methylation level of m^6^A in the hippocampus of 5 × FAD mice. The results indicated that the overexpression of METTL16 led to an increase in the expression levels of both MAT2A protein and mRNA, as well as an elevation in the m^6^A methylation level of MAT2A mRNA. This, in conjunction with previously observed METTL16 overexpression, resulted in an increased m^6^A level in the mouse hippocampus. We hypothesized that METTL16 might influence the m^6^A methylation level in the hippocampus of 5 × FAD mice by modulating the m^6^A methylation status of MAT2A mRNA, thereby impacting learning and memory, synaptic plasticity, and the expression level of Aβ_1–42_ in these mice. In this study, concurrent overexpression of METTL16 and knockdown of MAT2A impaired learning and memory, disrupted synaptic plasticity, and elevated the expression level of Aβ_1–42_ in 5 × FAD mice, consistent with findings from previous research.

In conclusion, the current study demonstrated that METTL16 enhances learning and memory in 5 × FAD mice by regulating MAT2A mRNA m^6^A methylation, which in turn increases the expression levels of PSD95 and Syp, enhances dendritic spine density, and reduces Aβ_1–42_ accumulation in the hippocampus. These findings reveal a novel approach for investigating the pathophysiological role of METTL16 in AD and offer new insights for the development of potential therapeutic targets for AD.

## Data Availability

The original contributions presented in the study are included in the article/supplementary material, further inquiries can be directed to the corresponding authors.
